# Risk Assessment and Prediction of Heavy Metal Pollution in Groundwater and River Sediment: A Case Study of a Typical Agricultural Irrigation Area in Northeast China

**DOI:** 10.1155/2015/921539

**Published:** 2015-08-23

**Authors:** Shuang Zhong, Hui Geng, Fengjun Zhang, Zhaoying Liu, Tianye Wang, Boyu Song

**Affiliations:** ^1^Key Lab of Groundwater Resources and Environment, Ministry of Education, Jilin University, Changchun 130021, China; ^2^Environmental Assessment Center of Jilin Province, Changchun 130022, China; ^3^Environmental Protection Research Institute of Light Industry, Beijing 100089, China

## Abstract

The areas with typical municipal sewage discharge river and irrigation water function were selected as study sites in northeast China. The samples from groundwater and river sediment in this area were collected for the concentrations and forms of heavy metals (Cr(VI), Cd, As, and Pb) analysis. The risk assessment of heavy metal pollution was conducted based on single-factor pollution index (*I*) and Nemerow pollution index (NI). The results showed that only one groundwater sampling site reached a polluted level of heavy metals. There was a high potential ecological risk of Cd on the N21-2 sampling site in river sediment. The morphological analysis results of heavy metals in sediment showed that the release of heavy metals can be inferred as one of the main pollution sources of groundwater. In addition, the changes in the concentration and migration scope of As were predicted by using the Groundwater Modeling System (GMS). The predicted results showed that As will migrate downstream in the next decade, and the changing trend of As polluted areas was changed with As content districts because of some pump wells downstream to form groundwater depression cone, which made the solute transfer upstream.

## 1. Introduction

Heavy metals pollution in water, soils, and sediment is increasingly serious in China along with the rapid industrialization and urbanization [1, 2]. Heavy metals as a type of persistent toxic pollutants [3] are unbiodegradable in the environment. Thus, the residual heavy metals in environment are threatening human health and ecosecurity [4, 5]. The main sources of heavy metals in the water are atmospheric precipitation, discharge of industrial wastewater and urban sewage, mineral mining, and infusion of surface runoff [6, 7]. Heavy metals are insoluble in the receiving water [8], and the majority of them are transformed from the aqueous phase to the solid phase and finally deposit in the sediment [9]. Due to this process, the contents of heavy metals in sediments were higher than those in aqueous phase; hence, that can be regarded as the accumulation library of heavy metals [10]. However, the heavy metals in the sediments can be released into the environment again [11], causing secondary pollution of the water [12] and chronically damaging the ecoenvironment [13, 14]. The heavy metals as nondegradable toxic substances in the water [15] can be enriched via food chains from low- to high-level organisms [16]. Such enrichment leads to direct or indirect accumulation of heavy metals in human body, causing chronic poisoning and threatening human health or even life [5, 17, 18].

The rapid economic development in northeast China has led to the quick increase of urban water demand and the expanding discharge of industrial wastewater and sewage, polluting the water system there severely.

This paper aims to investigate the heavy metal pollution in groundwater and the sediments, and the spatial distribution characterization of the heavy metals in water system. The risks of heavy metal pollution in groundwater and the degrees and potential ecological risks of heavy metal pollution in sediments were assessed. The migration scope of As in water system was predicted by using the Groundwater Modeling System (GMS).

## 2. Sample Collection and Measurement

The study area was located south of northeast China. The study area was dominated by croplands along the river, and the elevation (mean 45–50 m) generally declined from northeast to southwest. Most of the study areas were covered by quaternary loose deposits. The river water runs from northeast to southwest.

**Figure 1:**
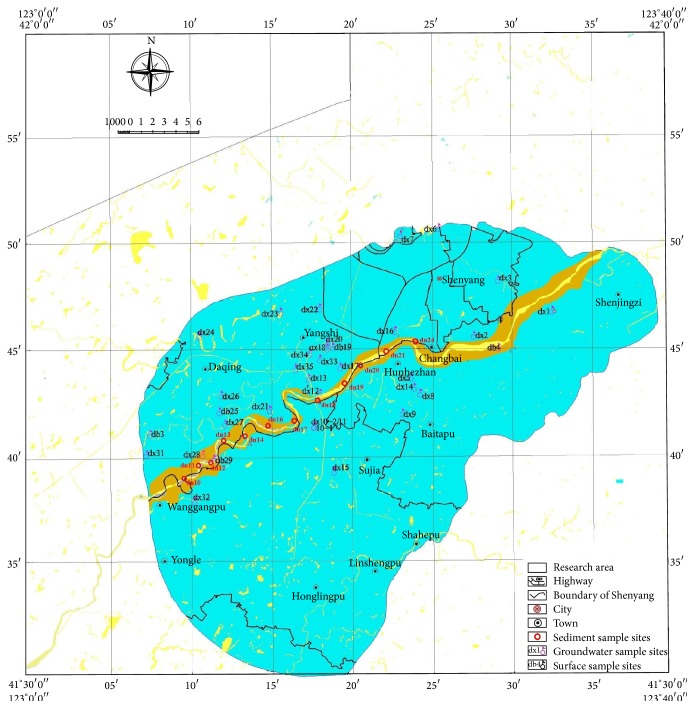
The distribution of the groundwater and sediment sampling sites.

Totally 33 groundwater sampling sites were distributed along the water flow direction of the river. Totally 12 sampling sites of sediment were distributed from 123°10′ to 123°28′E, with reference to the sampling sites of groundwater. The sediment at varying depth was stratified and sampled (31 samples). The distribution of the sampling sites is showed in Figure 1.

Cr, As, Cd, and Pb were selected as monitoring factors based on the historical research data of environment pollution in the study area as well as the tasks of this study. Water samples were filtered through a 0.45 *μ*m cellulose acetate membrane before measurement. Samples of sediments were dried in an oven at 105°C for 24 h and the samples were microwave digested with nitric acid, hydrochloric acid, hydrofluoric acid, and hydrogen peroxide before determination, as described in a previous study [19]. The contents of heavy metals (Cr(VI), As, Cd, and Pb) in groundwater samples and in sediment samples digested were measured using an inductively coupled plasma mass spectrometer (ICP-MS). Analytical quality control included analysis of reagent blank, sample blank, reference material, and three parallel samples.

## 3. Risk Assessment of Groundwater Pollution

### 3.1. Heavy Metal Pollution in Groundwater

**Figure 2:**
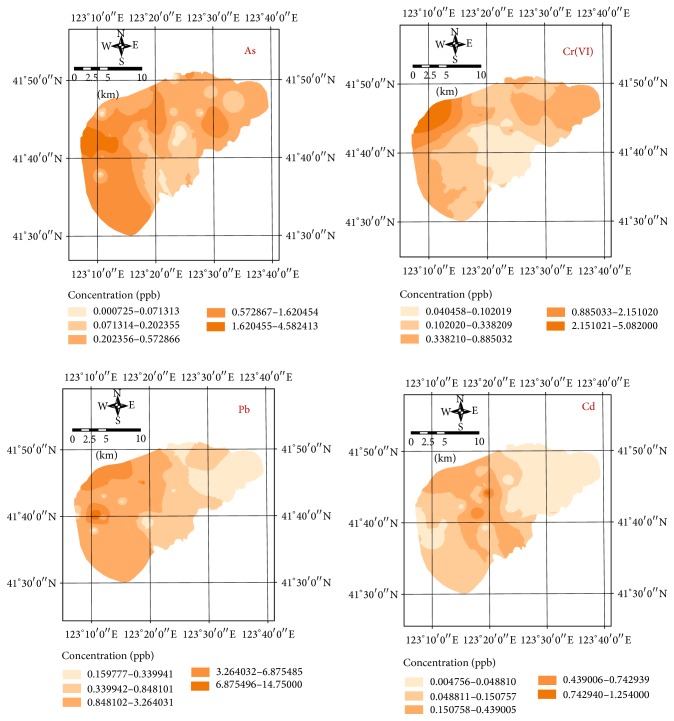
Distributions of As, Cr(VI), Pb, and Cd contents in groundwater.

The content distributions of Cr(VI), As, Cd, and Pb in groundwater were plotted in Figure 2 using ArcGIS according to the test results. Cr(VI), As, Cd, and Pb pollution in groundwater was assessed on basis of Chinese National Quality Standard for Groundwater (GB/T 14848-93).

Clearly, the As, Cr(VI), and Cd contents in groundwater did not exceed the thresholds in* Sanitary Standard for Drinking Water* (GB 5749-2006), and the contents of heavy metals in most sites met class I in GB/T 14848-93 (Figure 2). However, the Pb contents in some sampling sites exceeded the thresholds in the above two standards.

### 3.2. Risk Assessment of Heavy Metal Pollution in Groundwater

Two kinds of risk assessment models of heavy metal pollution degree were used to evaluate the heavy metal pollution in the groundwater in the study area.

#### 3.2.1. Single-Factor Pollution Index (*I*)

Single-factor pollution index (*I*) is used to assess how a single heavy metal pollutes groundwater at a sampling site:

(1) $$\begin{array}{c} I_{i}=\frac{C_{i}}{S_{i}}, \end{array}$$

where *C*_*i*_ is the measured content of pollutant *i* in surface water (*μ*m·L^−1^) and *S*_*i*_ is the evaluation standard of pollutant *i* in surface water (*μ*m·L^−1^). When *I*_*i*_ is >1, the content of that heavy metal exceeds the standard [20].

**Figure 3:**
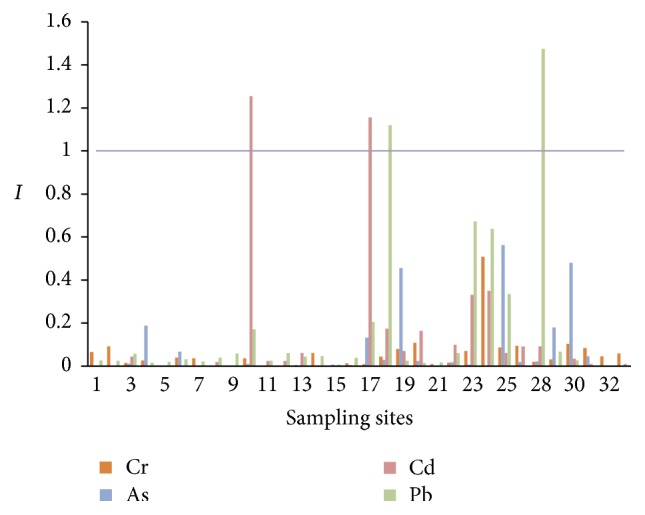
Single-factor pollution indices of heavy metals at all groundwater sampling sites.

The results of single-factor pollution index of heavy metals in groundwater in the study areas are showed in Figure 3 using the class II standards in GB/T 14848-93. The *I*s of Cd at sites 10 and 17 were >1, indicating Cd contents exceeded the standard. The Pb contents at sites 18 and 28 also exceeded the standard. The *I*s of Cr(VI) and As did not exceed 1, indicating the contents of these two heavy metals met the class II standards in GB/T 14848-93.

#### 3.2.2. Nemerow Pollution Index

Nemerow pollution index (NI) is used to assess how several heavy metals pollute groundwater at a sampling site. This index considers the mean and maximum values of single-factor pollution index and highlights the pollutants with high pollution degrees. It is expressed as

(2) $$\begin{array}{c} \text{NI}={\left(\;\frac{{\left[\left(1/n\right)\sum \left(C_{i}/S_{i}\right)\right]}^{2}+{\left[\max\left(C_{i}/S_{i}\right)\right]}^{2}}{2}\;\right)}^{1/2}, \end{array}$$

where *n* is the number of indices; *C*_*i*_ is the measured content of heavy metal *i*; *S*_*i*_ is the standard value. The heavy metal pollution in groundwater is divided by NI into 6 degrees [21] (Table 1).

The pollution degree and pollution level of heavy metals in groundwater were assessed with NI.

**Figure 4:**
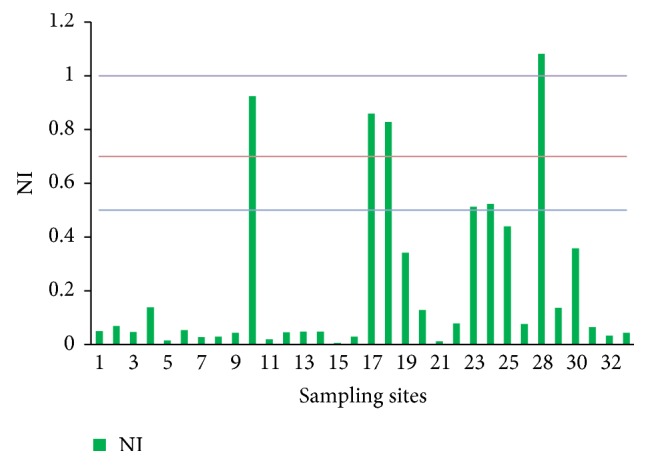
NIs of heavy metals at all groundwater sampling sites.

The heavy metal pollution was evaluated using class II standard in GB/T 14848-93. The NIs of heavy metals in groundwater in the study area are showed in Figure 4. The NI at site 28 exceeded the standard; the NIs at sites 10, 17, and 18 were at warming level. The NIs at other sites were all at clean or no-pollution level.

### 3.3. Prediction of Long-Term Risks of Groundwater Pollution

The solute transport of As was simulated by using GMS with the above models and the results, under the natural state without adding any external factors such as anthropogenic impact. The forms and distribution of pollution halo were characterized and the contents of specific pollutants in groundwater were predicted.

#### 3.3.1. Simulation of Solute Transport under Initial Conditions

**Figure 5:**
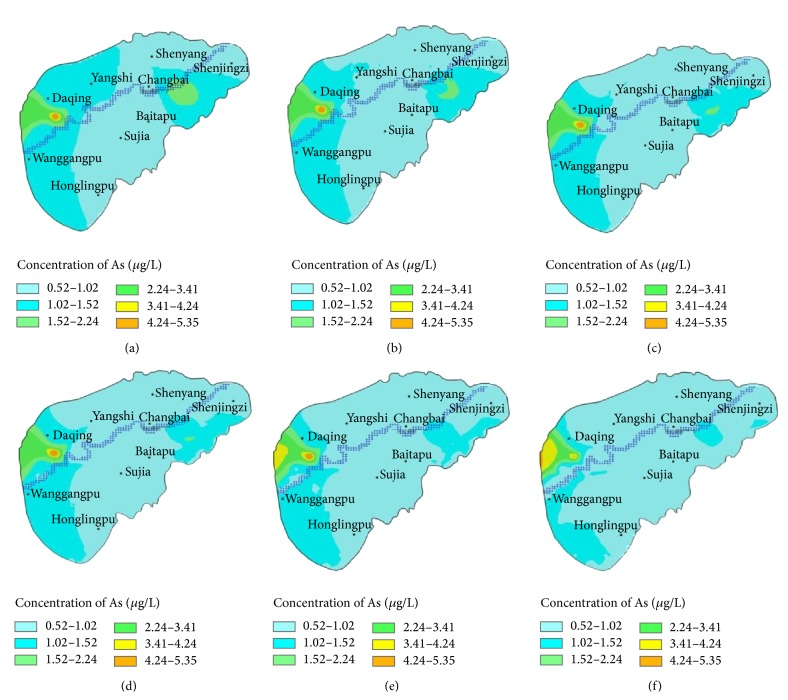
Diffusion and transport of As in shallow aquifer. (a) Distribution of initial content; (b) after 1 year; (c) after 2 years; (d) after 3 years; (e) after 5 years; (f) after 10 years.

The solute transport was simulated by using the measured As contents as initial conditions without considering other pollution sources. The simulated As transport at different time periods in the study area is illustrated in Figure 5. The distribution area of As at different concentration ranges in different years was calculated by using the ArcGIS software.

The simulated results showed that the As polluted area was 286.28 km^2^ in the west of the study area and 98.36 km^2^ in the east of the study area at the moment, which indicated the main polluted area was in the downstream of the Hun River. We will discuss the west part mainly. The polluted areas were divided into five districts according to the As contents. The highest As content in the center was 4.25 *μ*g/L in the west polluted area with 1.02 km^2^; the As content in the second district was 3.5 *μ*g/L with 2.28 km^2^, followed by the As content being 2.25 *μ*g/L with 23.41 km^2^, 1.5 *μ*g/L with 38.91 km^2^, and 1.0 *μ*g/L with 286.28 km^2^, respectively. The contents and the areas after As transfer in different years were showed in Table 2. The results showed that the polluted areas were decreased gradually for the 10-year transfer in low content districts and decreased gradually for first 5-year transfer and increased gradually for last 5-year transfer in high content districts. After 10 years of transfer, the As polluted areas were 2.25 km^2^ with 4.25 *μ*g/L, 12.63 km^2^ with 3.5 *μ*g/L, 24.22 km^2^ with 2.25 *μ*g/L, 36.13 km^2^ with 1.5 *μ*g/L, and 119.30 km^2^ with 1.0 *μ*g/L, respectively. The reason why the As polluted areas were increased in high content districts because these were some pump wells downstream, which pumped about 2000 m^3^/d each well and made the solute transfer upstream because of the funnels formed.

#### 3.3.2. Simulation of Solute Transport with Addition of Pollution Sources

**Figure 6:**
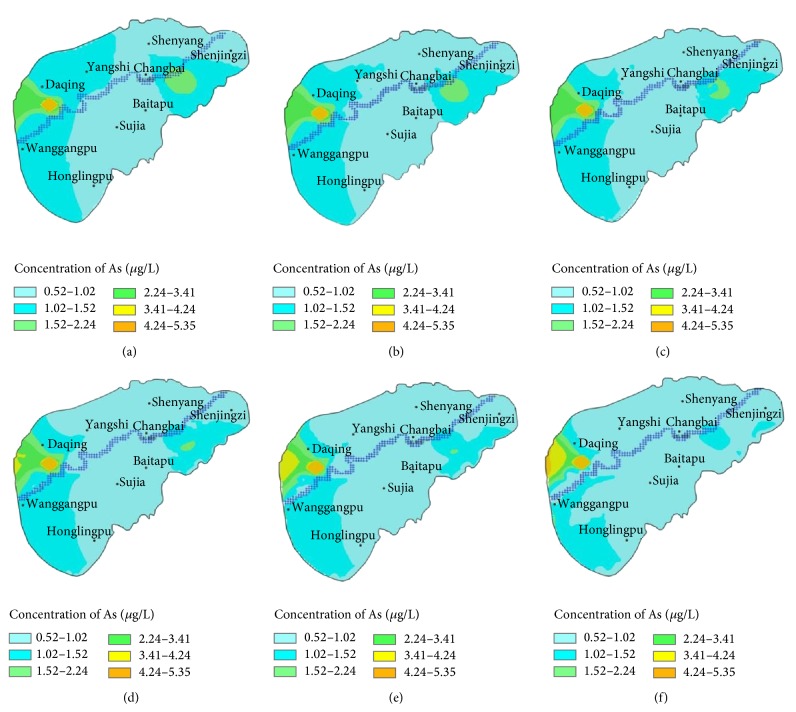
Diffusion and transport of As in shallow aquifer with addition of a pollution source. (a) Distribution of initial content; (b) after 1 year; (c) after 2 years; (d) after 3 years; (e) after 5 years; (f) after 10 years.

Into the severely polluted areas, a continuous pollution source with constant pollution concentration was added and the solute transfer was simulated. The simulated results were showed in Figure 6.

With the addition of a pollution source, the simulated results showed that As transfer areas did not change largely from those under the initial conditions; the As polluted area was 286.28 km^2^ in the west of the study area at the moment. The highest As content in the center was 4.25 *μ*g/L in the west polluted area with 3.82 km^2^, 3.5 *μ*g/L with 4.63 km^2^, 2.25 *μ*g/L with 24.88 km^2^, 1.5 *μ*g/L with 42.04 km^2^, and 1.0 *μ*g/L with 295.09, respectively. The contents and the areas after As transfer in different years were showed in Table 3. The results showed that the changing trend of polluted areas was almost the same with those as initial conditions. However, the highest As content area was not changed almost because of the addition of a pollution source. After 10 years of transfer, the As polluted areas were 3.23 km^2^ with 4.25 *μ*g/L, 14.24 km^2^ with 3.5 *μ*g/L, 27.31 km^2^ with 2.25 *μ*g/L, 35.94 km^2^ with 1.5 *μ*g/L, and 135.80 km^2^ with 1.0 *μ*g/L, respectively.

## 4. Risk Assessment of Heavy Metal Pollution in Sediments

### 4.1. Contents of Heavy Metals in Sediment

Sediment is a key place in the water suitable for accumulation of heavy metals. The contents of heavy metals in sediment reflect the degree of heavy metal pollution in the water. To clarify the sources of heavy metals in groundwater, we also collected sediment at varying depth. The contents of Cr(VI), As, Cd, and Pb were measured using ICP-MS. The results are showed in Table 4.

Clearly, Cr pollution was the most severe in sediment of Hun River, and the Cr contents in most samples exceeded the background value, followed by Pb. Cd content only at site N21-2 exceeded the background value, and no As content exceeded the background value.

### 4.2. Risk Assessment of Heavy Metal Pollution in Sediments

#### 4.2.1. Risk Assessment of Pollution Degree

Single-factor pollution index *C*_*f*_^*i*^ is expressed as follows:

(3) $$\begin{array}{c} C_{f}^{i}=\frac{C_{D}^{i}}{C_{R}^{i}}, \end{array}$$

where *C*_*D*_^*i*^ is the measured heavy metal content in sediment and *C*_*R*_^*i*^ is the background content of a heavy metal (Table 4). *C*_*f*_^*i*^ > 1 indicates “pollution” of a heavy metal, and *C*_*f*_^*i*^ < 1 indicates a “clean” state (Table 5).

Compound pollution index (CPI) was used to assess the heavy metal pollution in sediment, which is expressed as follows:

(4) $$\begin{array}{c} \text{CPI}=\overset{m}{\underset{i=1}{\sum }}\frac{C_{f}^{i}}{m}, \end{array}$$

where *C*_*f*_^*i*^ is a single-factor index of a heavy metal and *m* is the number of heavy metal types. CPI < 1 indicates no heavy metal pollution in sediment; CPI ≥ 1 indicates heavy metal pollution. The results were showed in Table 5.

The pollution levels of the heavy metal were clarified in Table 6. Based on these results, we can see that Cr pollution was the most severe in the river sediment, and the Cr contents in most sampling sites exceeded the background value, followed by Pb (Tables 5-6). Cd content only at site N21-2 exceeded the background value, and no As content exceeded the background value. The sampling sites N12-1, N19-2, N21-2, and S20-1 were at comprehensive pollution, and in particular, the CPI of N21-2 was 2.1716.

#### 4.2.2. Risk Assessment of Heavy Metal Potential Ecological Risks in Sediment

The potential ecological risk index of a heavy metal, *E*_*r*_^*i*^, is expressed as follows [20]:

(5) $$\begin{array}{c} E_{r}^{i}=T_{r}^{i}\times C_{f}^{i}, \end{array}$$

where *T*_*r*_^*i*^ is the toxicity response coefficient of that heavy metal and reflects the toxicity of the heavy metal and the water body's sensitivity to the heavy metal pollution; *C*_*f*_^*i*^ is the coefficient of pollution.

The potential ecological risk index of multiple heavy metal (RI) is expressed as follows [20]:

(6) $$\begin{array}{c} \text{RI}=\overset{m}{\underset{i=1}{\sum }}E_{r}^{i}. \end{array}$$

The ecological risk of the study area was assessed using the classification of all potential ecological risk indices (Table 7).

Clearly, *E*_*r*_^*i*^ of four heavy metals was <40, indicating the four heavy metals were at low potential ecological risks. The RI at site N21-2 was 40–80, indicating severe risk. The other sampling sites were at low potential ecological risks.

### 4.3. Morphologic Analysis of Heavy Metals in Sediment

The major states of heavy metals include weak acid-extractable form, reducible form, oxidizable form, and residual form [22]. We analyzed the forms of heavy metals at the severely polluted sites (N19-2, S20-1, N21-1, and N21-2) according to* Soil and Sediment—Sequential Extraction Procedure of Speciation of 13 Trace Elements* (GB/T25282-2010). The results are showed in Table 8.

Clearly, the proportions of bioavailability forms of Cd were all the highest among the four metals at four sites, especially the weak acid-extractable form. The forms indicate that Cd is most soluble in rivers and its migration features facilitate its migration via the water into groundwater, which is basically consistent with the heavy metal pollution situations in groundwater described in Section 3.1. Then Pb was mainly in the reducible form, indicating that Pb can be easily released into the environment to pollute the water. Therefore, we deduce that the release of heavy metals from sediment is a major pollution source of heavy metals in groundwater.

## 5. Conclusions

(1) The comprehensive pollution at groundwater site 28 reached the pollution level, sites 10, 17, and 18 reached warning level, and other sites were all clean. The changes and migration scope of As content indicate that As migrated downstream to Hun River in recent 10 years; the polluted areas were decreased gradually in low content districts and decreased gradually first and increased gradually later in high content districts because these were some pump wells downstream to form groundwater depression cone, which made the solute transfer upstream.

(2) Both the Cr(VI) and Pb contents in most sediment samples exceeded the national background values. The As contents at all sites did not exceed the background value. The potential ecological risk of Cd was very high at site N21-2, but not at other sites.

(3) Through the morphological analysis of heavy metals at four sites where the contents were the highest, the proportions of biodegradable form of Cd were all the highest among the four metals at four sites, secondly was the weak acid-extractable, which all indicated Cd could be easily released into the water and polluted the groundwater due to the migration. Pb was mainly in the reduction form, indicating that Pb also can be released into the environment and pollute the water. Therefore, we deduce that the release from sediment is a major source of heavy metal pollution in groundwater.

## Figures and Tables

**Table 1:** Classification of NI.

Class	Pollution degree	NI
0	No pollution	≤0.5
1	Clean	0.5–0.7
2	Warm	0.7–1.0
3	Polluted	1.0–2.0
4	Medium pollution	2.0–3.0
5	Severe pollution	>3.0

**Table 2:** Distribution area of As at different concentration ranges in different years.

Time (year)	Distribution area (km^2^)				
	*C* > 1.0 *μ*g/L	*C* > 1.5 *μ*g/L	*C* > 2.25 *μ*g/L	*C* > 3.5 *μ*g/L	*C* > 4.25 *μ*g/L
0	286.28	38.91	23.41	2.28	1.02
1	229.15	41.12	24.43	2.47	1.00
2	210.34	42.04	25.46	2.66	0.96
3	187.49	42.27	26.29	6.10	0.95
5	177.79	42.46	26.52	10.44	0.74
10	119.30	36.13	24.23	13.60	2.28

**Table 3:** Distribution area of As at different concentration ranges in different years with addition of a pollution source.

Time (year)	Distribution area (km^2^)				
	*C* > 1.0 *μ*g/L	*C* > 1.5 *μ*g/L	*C* > 2.25 *μ*g/L	*C* > 3.5 *μ*g/L	*C* > 4.25 *μ*g/L
0	295.09	42.04	24.88	4.63	3.83
1	242.75	43.21	25.69	4.67	3.84
2	220.01	44.06	26.53	4.76	3.89
3	212.73	45.01	27.52	3.51	3.99
5	228.14	50.28	30.65	13.38	4.77
10	135.80	35.95	27.31	14.24	3.23

**Table 4:** Contents of heavy metals in sediment of Hun River (ppm).

Number	Thickness (cm)	Cr(VI)	As	Cd	Pb
N10-1	0–26	62.58 ± 0.04	3.51 ± 0.13	0.12 ± 0.03	15.29 ± 0.12
N10-2	26–40	47.16 ± 0.06	2.26 ± 0.12	0.04 ± 0.02	12.48 ± 0.10
N11-1	0–25	114.10 ± 0.02	7.15 ± 0.16	0.25 ± 0.04	37.47 ± 0.09
N11-2	25–45	98.22 ± 0.10	5.56 ± 0.06	0.20 ± 0.06	29.85 ± 0.16
N11-3	45–50	60.41 ± 0.11	3.92 ± 0.05	0.12 ± 0.04	21.45 ± 0.12
N12-1	0–50	147.30 ± 0.08	4.71 ± 0.07	0.31 ± 0.06	77.98 ± 0.13
N13-1-1	0–5	90.11 ± 0.06	4.93 ± 0.07	0.18 ± 0.03	33.43 ± 0.11
N13-1-2	5–30	21.93 ± 0.02	1.46 ± 0.11	0.02 ± 0.02	11.14 ± 0.17
N13-2-1	0–15	68.71 ± 0.05	4.25 ± 0.08	0.20 ± 0.04	21.90 ± 0.05
N13-2-2	15–40	111.30 ± 0.11	7.61 ± 0.07	0.18 ± 0.06	30.26 ± 0.06
N13-2-3	40–64	111.10 ± 0.09	6.11 ± 0.04	0.29 ± 0.08	30.43 ± 0.10
N14-1	0–23	70.67 ± 0.05	5.02 ± 0.05	0.20 ± 0.04	19.53 ± 0.06
N14-2	23–30	77.33 ± 0.12	6.14 ± 0.08	0.18 ± 0.06	23.94 ± 0.08
N14-3	30–40	105.50 ± 0.11	8.36 ± 0.08	0.28 ± 0.08	40.66 ± 0.11
N14-4	40–50	39.99 ± 0.08	3.45 ± 0.07	0.11 ± 0.08	14.68 ± 0.05
N16-1	0–26	60.38 ± 0.04	3.65 ± 0.06	0.14 ± 0.07	19.98 ± 0.05
N16-2	26–50	58.30 ± 0.06	3.92 ± 0.06	0.09 ± 0.07	20.92 ± 0.07
N17	0–32	58.03 ± 0.04	1.87 ± 0.03	0.06 ± 0.05	15.06 ± 0.07
N18	0–28	113.70 ± 0.09	3.86 ± 0.11	0.18 ± 0.06	45.28 ± 0.07
N19-1	0–5	87.94 ± 0.11	2.80 ± 0.05	0.28 ± 0.07	19.42 ± 0.06
N19-2	5–25	141.40 ± 0.10	6.55 ± 0.06	0.28 ± 0.11	61.48 ± 0.11
N20-1	0–29	116.50 ± 0.13	3.89 ± 0.04	0.33 ± 0.14	42.30 ± 0.10
N21-1	0–5	97.22 ± 0.04	4.40 ± 0.03	0.20 ± 0.07	58.54 ± 0.07
N21-2	5–26	197.80 ± 0.09	12.84 ± 0.07	0.64 ± 0.04	142.60 ± 0.14
N24-1	Surface layer	35.64 ± 0.04	2.32 ± 0.02	0.07 ± 0.05	15.65 ± 0.05
S14-1	Surface layer	74.07 ± 0.05	4.69 ± 0.04	0.15 ± 0.08	24.43 ± 0.06
S16-1	Surface layer	17.23 ± 0.03	1.17 ± 0.02	0.03 ± 0.03	9.46 ± 0.05
S19-1	0–44	86.18 ± 0.07	11.61 ± 0.06	0.19 ± 0.05	35.57 ± 0.08
S19-2	0–44	83.73 ± 0.08	12.21 ± 0.05	0.21 ± 0.05	27.86 ± 0.09
S20-1	0–13	150.20 ± 0.11	6.97 ± 0.06	0.38 ± 0.08	71.41 ± 0.07
S20-2	13–22	73.72 ± 0.08	4.21 ± 0.06	0.10 ± 0.07	26.27 ± 0.14

Background value		60	15	0.5	35

Note: the data are the mean ± standard deviation.

**Table 5:** Assessment of heavy metal pollution degree risks in sediment.

Number	Thickness (cm)	*C*_*f*_^*i*^				CPI
		*C*_*f*_^Cr^	*C*_*f*_^As^	*C*_*f*_^Cd^	*C*_*f*_^Pb^	
N10-1	0–26	0.78	0.23	0.24	0.44	0.42
N10-2	26–40	0.59	0.15	0.07	0.36	0.29
N11-1	0–25	1.43	0.48	0.51	1.07	0.87
N11-2	25–45	1.23	0.37	0.40	0.85	0.71
N11-3	45–50	0.76	0.26	0.24	0.61	0.47
N12-1	0–50	1.84	0.31	0.62	2.23	1.25
N13-1-1	0–5	1.13	0.33	0.36	0.96	0.69
N13-1-2	5–30	0.27	0.10	0.05	0.32	0.18
N13-2-1	0–15	0.86	0.28	0.39	0.63	0.54
N13-2-2	15–40	1.39	0.51	0.37	0.87	0.78
N13-2-3	40–64	1.39	0.41	0.59	0.87	0.81
N14-1	0–23	0.88	0.34	0.40	0.56	0.54
N14-2	23–30	0.97	0.41	0.36	0.68	0.61
N14-3	30–40	1.32	0.56	0.56	1.16	0.90
N14-4	40–50	0.50	0.23	0.23	0.42	0.34
N16-1	0–26	0.76	0.24	0.27	0.57	0.46
N16-2	26–50	0.73	0.26	0.18	0.60	0.44
N17	0–32	0.73	0.13	0.11	0.43	0.35
N18	0–28	1.42	0.26	0.35	1.29	0.83
N19-1	0–5	1.10	0.19	0.57	0.56	0.60
N19-2	5–25	1.77	0.44	0.56	1.76	1.13
N20-1	0–29	1.46	0.26	0.67	1.21	0.90
N21-1	0–5	1.22	0.29	0.40	1.67	0.90
N21-2	5–26	2.47	0.86	1.28	4.07	2.17
N24-1	Surface layer	0.45	0.15	0.14	0.45	0.30
S14-1	Surface layer	0.93	0.31	0.31	0.70	0.56
S16-1	Surface layer	0.22	0.08	0.05	0.27	0.15
S19-1	0–44	1.08	0.77	0.38	1.02	0.81
S19-2	0–44	1.05	0.81	0.42	0.80	0.77
S20-1	0–13	1.88	0.46	0.77	2.04	1.29
S20-2	13–22	0.92	0.28	0.21	0.75	0.54

**Table 6:** Heavy metal pollution levels.

Number	Pollution level				Comparison
	Cr	As	Cd	Pb	
N11-1	Polluted	—	—	Polluted	Cr > Pb
N11-2	Polluted	—	—	—	
N12-1	Polluted	—	—	Polluted	Cr > Pb
N13-1-1	Polluted	—	—	Polluted	
N13-2-2	Polluted	—	—	—	
N13-2-3	Polluted	—	—	—	
N18	Polluted	—	—	Polluted	Cr > Pb
N19-1	Polluted	—	—	—	
N19-2	Polluted	—	—	Polluted	Cr > Pb
N20-1	Polluted	—	—	Polluted	Cr > Pb
N21-1	Polluted	—	—	Polluted	Cr > Pb
N21-2	Polluted	—	Polluted	Polluted	Pb > Cr > Cd
S19-1	Polluted	—	—	Polluted	Cr > Pb
S19-2	Polluted	—	—	—	
S20-1	Polluted	—	—	Polluted	Pb > Cr

**Table 7:** Assessment of heavy metal potential ecological risks in sediment.

Number	Thickness (cm)	*E*_*r*_^*i*^				RI
		Cr	As	Cd	Pb	
N10-1	0–26	1.18	1.50	2.20	1.78	6.67
N10-2	26–40	1.56	2.34	7.10	2.18	13.19
N11-1	0–25	2.85	4.77	15.25	5.35	28.23
N11-2	25–45	2.46	3.70	12.11	4.26	22.54
N11-3	45–50	1.51	2.61	7.08	3.06	14.27
N12-1	0–50	3.68	3.14	18.59	11.14	36.55
N12-2	Surface layer	2.20	3.58	9.58	3.69	19.05
N13-1-1	0–5	2.25	3.29	10.71	4.78	21.02
N13-1-2	5–30	0.55	0.98	1.41	1.59	4.53
N13-2-1	0–15	1.72	2.83	11.72	3.13	19.40
N13-2-2	15–40	2.78	5.07	10.96	4.32	23.14
N13-2-3	40–64	2.78	4.08	17.55	4.35	28.75
N14-1	0–23	1.77	3.35	11.99	2.79	19.90
N14-2	23–30	1.93	4.09	10.78	3.42	20.22
N14-3	30–40	2.64	5.57	16.70	5.81	30.72
N14-4	40–50	1.00	2.30	6.79	2.10	12.19
N16-1	0–26	1.51	2.43	8.12	2.85	14.92
N16-2	26–50	1.46	2.61	5.31	2.99	12.36
N17	0–32	1.45	1.25	3.34	2.15	8.19
N18	0–28	2.84	2.57	10.55	6.47	22.43
N19-1	0–5	2.20	1.86	17.09	2.77	23.93
N19-2	5–25	3.54	4.36	16.85	8.78	33.53
N20-1	0–29	2.91	2.60	20.02	6.04	31.57
N21-1	0–5	2.43	2.94	11.94	8.36	25.67
N21-2	5–26	4.95	8.56	38.51	20.37	72.39
N24-1	Surface layer	0.89	1.54	4.05	2.24	8.72
S14-1	Surface layer	1.85	3.13	9.16	3.49	17.63
S16-1	Surface layer	0.43	0.78	1.63	1.35	4.19
S19-1	0–44	2.15	7.74	11.51	5.08	26.49
S19-2	0–44	2.09	8.14	12.62	3.98	26.84
S20-1	0–13	3.76	4.64	22.97	10.20	41.57
S20-2	13–22	1.84	2.80	6.25	3.75	14.65

**Table 8:** Morphologic analysis of heavy metals in sediment.

Number	Heavy metals	Proportion of various forms %				Morphological distribution order
		A (weak acid-extractable form)	B (reducible form)	C (oxidizable form)	D (residual form)	
N19-2	Cr(VI)	0.11 ± 0.09	0.25 ± 0.17	0.90 ± 0.32	98.74 ± 0.36	D > C > B > A
	As	0.66 ± 0.12	0.91 ± 0.13	0.33 ± 0.17	98.10 ± 0.33	D > B > A > C
	Cd	6.88 ± 0.18	2.96 ± 0.17	1.33 ± 0.16	88.83 ± 0.27	D > A > B > C
	Pb	0.16 ± 0.11	1.45 ± 0.26	0.09 ± 0.08	98.30 ± 0.36	D > B > A > C

S20-1	Cr(VI)	0.53 ± 0.21	0.60 ± 0.24	3.98 ± 0.27	94.88 ± 0.55	D > C > B > A
	As	0.76 ± 0.23	1.56 ± 0.21	0.64 ± 0.21	97.04 ± 0.30	D > B > A > C
	Cd	5.85 ± 0.19	2.89 ± 0.17	0.70 ± 0.33	90.56 ± 0.26	D > A > B > C
	Pb	0.12 ± 0.08	1.60 ± 0.32	0.10 ± 0.08	98.18 ± 0.41	D > B > A > C

N21-1	Cr(VI)	0.15 ± 0.10	0.38 ± 0.21	2.62 ± 0.14	96.85 ± 0.27	D > C > B > A
	As	1.22 ± 0.14	0.95 ± 0.27	0.54 ± 0.17	97.28 ± 0.11	D > A > B > C
	Cd	22.74 ± 0.19	3.32 ± 0.33	1.34 ± 0.22	72.60 ± 0.12	D > A > B > C
	Pb	0.28 ± 0.11	2.30 ± 0.15	0.25 ± 0.19	97.17 ± 0.16	D > B > A > C

N21-2	Cr(VI)	0.08 ± 0.06	0.31 ± 0.12	0.75 ± 0.33	98.86 ± 0.27	D > C > B > A
	As	0.47 ± 0.21	1.07 ± 0.20	0.66 ± 0.25	97.80 ± 0.20	D > B > C > A
	Cd	5.49 ± 0.25	3.53 ± 0.25	4.97 ± 0.29	86.01 ± 0.11	D > A > C > B
	Pb	0.12 ± 0.08	1.74 ± 0.23	0.38 ± 0.16	97.77 ± 0.29	D > B > C > A

Note: the data are the mean ± standard deviation.
